# A Case of Peripheral Odontogenic Myxofibroma Arising in the Palatal Gingiva of the Maxillary Second Premolar Region: A Case Report

**DOI:** 10.1155/crid/7333467

**Published:** 2026-06-04

**Authors:** Masanori Masui, Hirokazu Yutori, Ai Fujimura, Soichiro Ibaragi

**Affiliations:** ^1^ Department of Oral and Maxillofacial Surgery, Faculty of Medicine, Dentistry and Pharmaceutical Sciences Okayama University, Okayama, Japan; ^2^ Department of Oral and Maxillofacial Surgery, Kagawa Prefectural Central Hospital, Takamatsu, Kagawa, Japan, chp-kagawa.jp

**Keywords:** case report, excisional biopsy, palatal gingiva, peripheral odontogenic myxofibroma, peripheral odontogenic myxoma

## Abstract

Odontogenic myxofibroma (OMF) is a rare benign mesenchymal odontogenic tumor characterized by myxoid stroma with a prominent fibrous component. Although it usually arises intraosseously within the jaws, the peripheral variant, peripheral odontogenic myxofibroma (POMF), which occurs in extraosseous soft tissues, is uncommon and may be clinically misdiagnosed as a reactive gingival lesion. We report a case of POMF in a 68‐year‐old man who was referred for evaluation of a painless, slowly enlarging swelling of the palatal gingiva in the left maxillary second premolar region, which had initially been diagnosed as chronic periodontitis at a local clinic. An intraoral examination revealed an elastic, firm mass with partial erythema on the palatal marginal gingiva. Panoramic radiography showed mild generalized horizontal bone loss without lesion‐specific changes, and computed tomography revealed no bone resorption associated with the lesion. Exfoliative cytology was negative for intraepithelial lesions or malignancy. The lesion was excised with a 5‐mm clinical margin, including periosteum, and superficial peripheral ostectomy of the adjacent cortical bone was performed. Histopathological examination revealed a myxoid stroma rich in mucinous matrix and collagen fibers, containing sparsely distributed spindle‐shaped cells and scattered nests of odontogenic epithelium. Alcian blue staining revealed diffuse positivity, supporting the diagnosis of POMF. No recurrence was observed during a 2‐year follow‐up period. This case highlights a diagnostic pitfall in the tooth‐bearing gingiva and underscores the importance of histopathological confirmation of persistent gingival masses. When imaging shows no apparent bone involvement, and clinical suspicion of malignancy is low, complete excision with an adequate soft‐tissue margin and selective, limited bone removal may achieve local control while preserving the adjacent teeth; long‐term follow‐up remains advisable.

## 1. Introduction

According to the 2024 World Health Organization (WHO) classification, odontogenic myxofibroma (OMF) is a benign mesenchymal odontogenic tumor. OMF is almost synonymous with odontogenic myxoma (OM); however, lesions that show a prominent fibrous component in addition to the myxoid component are often designated as OMF [1, 2].

Most cases of OM/OMF arise centrally within the jawbones. In contrast, its peripheral (extraosseous) counterpart, referred to as peripheral odontogenic myxoma (POM) or peripheral odontogenic myxofibroma (POMF), arises in gingival soft tissues, typically shows little or no bone involvement, and appears to have a lower recurrence rate than intraosseous lesions [3]. Because it presents as a small gingival mass in tooth‐bearing areas, POM/POMF can be mistaken for common reactive lesions or periodontal disease, resulting in delayed diagnosis or incomplete excision.

Herein, we report a case of a POMF arising in the palatal marginal gingiva of the left maxillary second premolar region that was initially managed as chronic periodontitis. This report emphasizes clinical diagnostic pitfalls and proposes a practical diagnostic strategy for persistent gingival masses, together with a literature review.

## 2. Case Report

A 68‐year‐old man was referred to our department for evaluation of swelling in the palatal gingiva of the left maxillary second premolar region. His medical history included hypertension and gastroesophageal reflux disease, and he was being treated with an angiotensin II receptor blocker. His family history was unremarkable.

**Figure 1:**
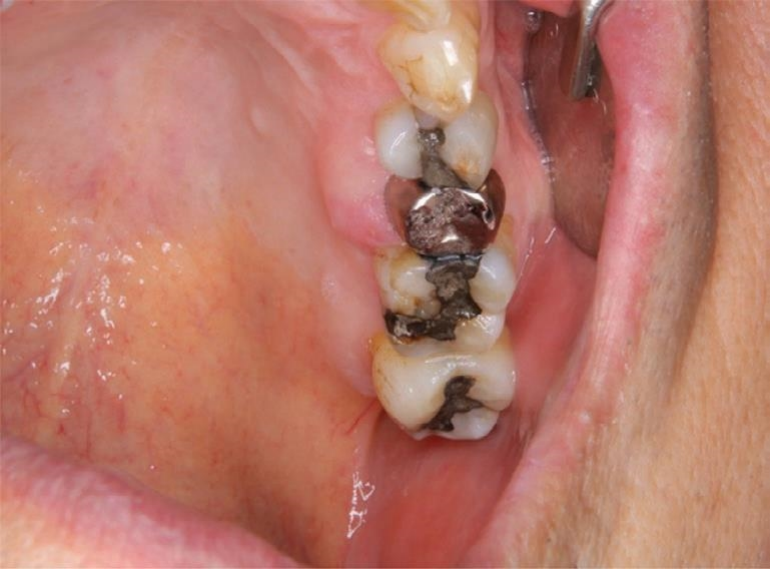
Intraoral photograph at initial presentation. A mass is present on the palatal aspect of the left maxillary second premolar with no extension into the buccal gingiva.

Six months prior to referral, swelling of the palatal gingiva adjacent to the left maxillary second premolar was noted during a routine dental checkup at a local clinic. The lesion was diagnosed as chronic periodontitis, and the patient was placed under observation. Because the swelling did not improve during the follow‐up visit, the patient was referred to our department for further examination and treatment. Intraoral examination revealed an elastic, firm mass with partial erythema in the palatal marginal gingiva of the left maxillary second premolar (Figure 1).

**Figure 2:**
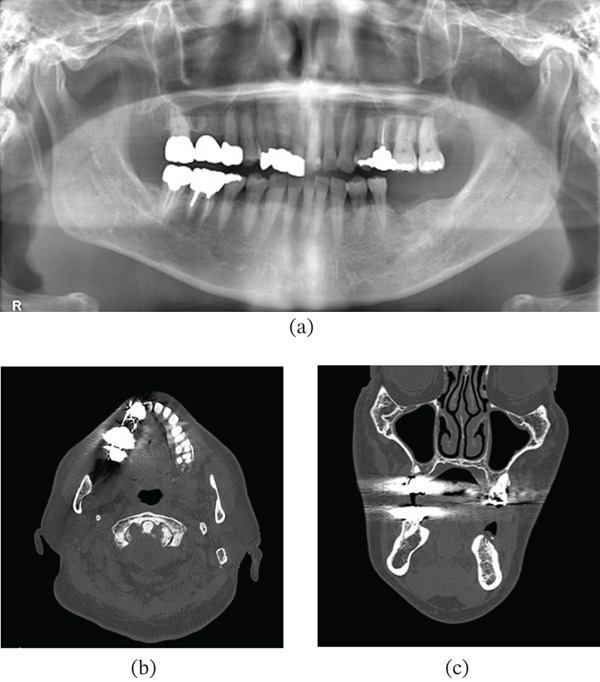
Imaging findings at initial presentation. (a) Orthopantomogram shows no lesion‐specific findings in the left maxillary second premolar region. Noncontrast computed tomography images ((b) axial; (c) coronal) show no resorption of the adjacent maxillary bone.

Panoramic radiography revealed mild generalized horizontal bone loss. Noncontrast‐enhanced computed tomography (CT) revealed no obvious bone resorption associated with the lesion (Figure 2). Based on these findings, a benign maxillary gingival tumor was provisionally diagnosed.

**Figure 3:**
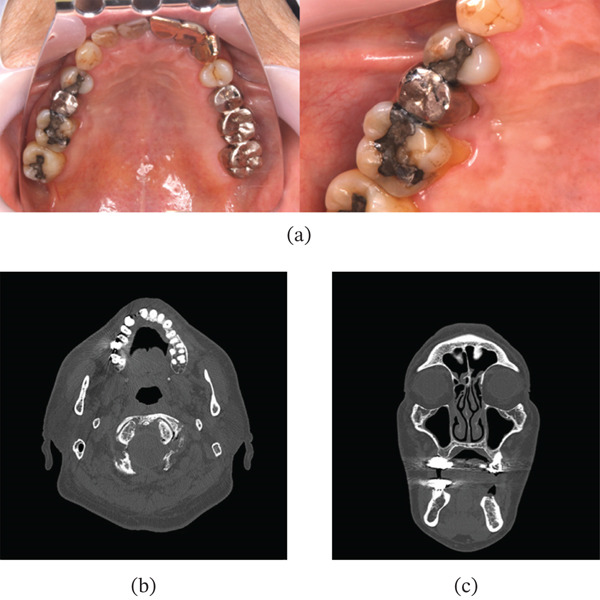
Postoperative intraoral photograph and CT images. (a) Intraoral photographs obtained 2 years postoperatively show no evidence of tumor recurrence. (b) Axial and (c) coronal CT images obtained 1 year postoperatively demonstrate only bone resorption attributable to peripheral ostectomy.

Exfoliative cytology performed at the initial visit revealed numerous enucleated keratinized squamous epithelial cells, and the findings were negative for intraepithelial lesions or malignancy (NILM). Given that the lesion was small, superficially located, showed no bone involvement on CT, and lacked clinical features suggestive of malignancy, we elected to perform an excisional biopsy to achieve both a definitive diagnosis and treatment in a single procedure. The tumor was surgically excised. The lesion was removed with a 5‐mm clinical margin in all directions, including the periosteum. The adjacent teeth were preserved, and the adjacent cortical bone was superficially removed using peripheral ostectomy. The postoperative course was uneventful. At the 2‐year follow‐up, there was no evidence of recurrence, and the clinical course remained favorable (Figure 3).

**Figure 4:**
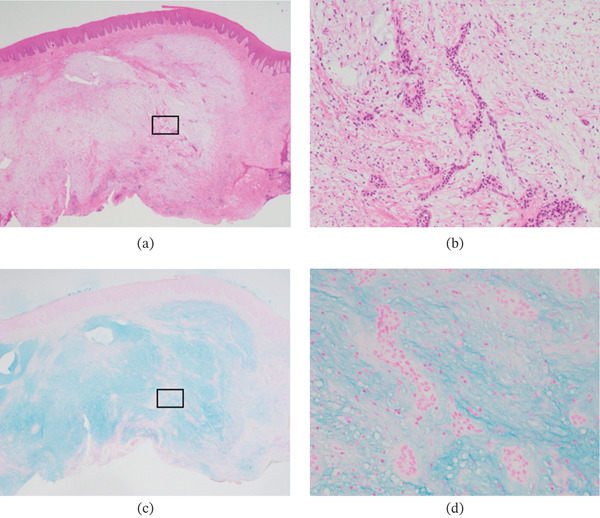
Histopathological findings. The mucosa is covered by stratified squamous epithelium, and spindle‐shaped cells are sparsely distributed within a myxoid stroma rich in mucinous matrix and collagen fibers. Small nests of odontogenic epithelium are scattered throughout the lesion, without marked nuclear atypia or increased mitotic activity (H&E staining: (a) ×12.5; (b) ×100). Alcian blue staining shows diffuse positivity throughout the subepithelial tissue, consistent with peripheral odontogenic myxofibroma ((c) ×12.5; (d) ×100).

Histopathological examination revealed that the mucosa was covered by stratified squamous epithelium. Spindle‐shaped cells were sparsely distributed within a myxoid stroma rich in mucinous matrix and collagen fibers. Small nests of odontogenic epithelium were scattered throughout the lesion, and no marked nuclear atypia or increased mitotic activity was observed. Alcian blue staining revealed diffuse positivity throughout the subepithelial tissue (Figure 4). Based on these findings, a diagnosis of POMF was made. A timeline summarizing the clinical course is presented in Table 1.

**Table 1:** Clinical timeline illustrating the course of this patient with peripheral odontogenic myxofibroma arising in the palatal gingiva of the maxillary second premolar region.

Date	Clinical progression
Six months earlier	Palatal gingival swelling near the left maxillary second premolar was noted at a routine check‐up and observed as chronic periodontitis.
Two weeks earlier	The swelling persisted at follow‐up.
First visit	An elastic hard palatal gingival mass was noted; panoramic radiography and CT showed no bone involvement, and exfoliative cytology was performed.
Twenty‐two days after	Excisional biopsy was performed with a 5‐mm margin including the periosteum, with limited peripheral ostectomy.
Twenty‐nine days after	Histopathological results were reported.
Two years after	No recurrence was observed.

## 3. Discussion

The reported incidence of OM ranges from 2.3% to 17.7% among all odontogenic tumors; when OMF is considered separately, its incidence is even lower [4]. Since 2000, only three cases of POMF, including the present case, have been reported. Even when POM, which is almost synonymous with POMF, is included, only 23 cases in 21 reports have been documented (Table 2) [3, 5–23]. Several clinically meaningful patterns can be drawn from Table 2. First, radiographic bone resorption is absent in many cases and, when present, tends to be superficial. Second, most cases are managed with local excision, and recurrence is uncommon when an adequate soft‐tissue margin is secured, with bone removal reserved for cases with suspected cortical involvement or for margin control. These patterns support a conservative, jaw‐preserving approach when cross‐sectional imaging confirms that the lesion is confined to the soft tissue; complete excision with a clinically adequate soft‐tissue margin and selective, limited bone removal (when indicated) may provide favorable local control while preserving the teeth and jaw structures. Although the small number of reported cases precludes definitive recommendations, the available data suggest that deep bony destruction is uncommon in peripheral lesions, supporting initial local management once malignancy is unlikely and bone involvement has been excluded.

**Table 2:** Reported cases of POM/POMF since 2000.

No.	Author	Year of report	Age (years)	Sex	Site of lesion		Tumor size (mm)	Configuration	Preoperative imaging	Bone resorption	Clinical diagnosis	Biopsy	Histopathological diagnosis	Odontogenic epithelium	Safety margin at surgery	Bone removal at resection	Follow‐up period (months)	Recurrence
1	Shimoyama et al. [5]	2000	51	M	Mandible	Labial gingiva of anterior region	30 × 28	Pedunculated	Occlusal radiograph	+	Peripheral ossifying fibroma	—	POM	+	Performed (details not available)	+	24	—
2	Chang et al. [6]	2001	37	M	Mandible	Lingual gingiva of molar region	15 × 12	Sessile	N/A	—	N/A	+	POM	—	N/A	—	8	—
3	Perrotti et al. [7]	2006	42	M	Maxilla	Anterior gingiva	10 × 10	Sessile	N/A	+	Fibroma	—	POM	—	N/A	—	48	—
4	Aytac‐Yazicioglu et al. [8]	2008	38	F	Maxilla	Buccal gingiva of molar region	30 × 20	Sessile	Panoramic radiograph	—	Peripheral giant cell granuloma	—	POM	+	Performed (details not available)	+	12	—
5	Raubenheimer and Noffke [3]	2012	53	F	Maxilla	Labial gingiva of anterior region	120 × 50	Pedunculated	Panoramic radiograph	+	Low‐grade sarcoma	+	POM	+	None	—	84	—
6			38	F	Mandible	Lingual gingiva of anterior region	80 × 60	Pedunculated	Panoramic radiograph	+	N/A	+	POM	+	N/A	—	N/A	—
7	Jain and Reddy [9]	2013	41	M	Maxilla	Labial gingiva of anterior region	20 × 10	Sessile	Panoramic radiograph	—	Fibroma	—	POM	+	N/A	—	6	—
8	Kapoor et al. [10]	2015	11	M	Infratemporal fossa		20 × 20	Unilocular	CT	—	N/A	+	POM	+	Tumor capsule	—	24	—
9	Tasnime et al. [11]	2016	14	F	Maxilla	Labial gingiva of anterior region	20 × 20	Pedunculated	Periapical radiograph	—	Peripheral giant cell granuloma	—	POM	—	None	+	9	—
10	Bhoyar et al. [12]	2016	8	F	Entire maxilla/mandible	Gingiva	N/A	Gingival enlargement	Panoramic radiograph	—	N/A	+	POM	—	N/A	N/A	N/A	N/A
11	Kanitkar et al. [13]	2017	12	F	Mandible	Anterior gingiva	15 × 10	Sessile	Occlusal radiograph	—	Pyogenic granuloma	—	POM	—	N/A	+	24	—
12	Bajpai and Pardhe [14]	2017	27	F	Maxilla	Labial gingiva of the anterior and premolar region	20 × 10	Pedunculated	Panoramic radiograph	+	Pyogenic granuloma	—	POM	—	N/A	—	12	—
13	Marco et al. [15]	2018	47	M	Mandible	Buccal gingiva of molar region	25 × 20	Sessile	Panoramic radiograph	+	Fibroma	—	POM	—	N/A	—	84	—
14			23	M	Mandible	Gingiva of the premolar alveolar ridge	10 × 5	Verrucous	Panoramic radiograph	+	Fibroma	—	POM	—	N/A	—	108	—
15	Sil [16]	2020	54	M	Mandible	Gingiva in the anterior and molar regions	30 × 20	Sessile	Panoramic radiograph	+	Mandibular gingival tumor	+	POMF	—	None	N/A	N/A	—
16	Albannai and Abosaleh [17]	2020	60	F	Maxilla	Labial gingiva of anterior region	20 × 20	Pedunculated	CT	+	N/A	+	POM	—	5 mm	+	12	—
17	Nakai et al. [18]	2021	55	M	Mandible	Lingual gingiva of anterior region	12 × 9	Sessile	CT, ultrasonography, and FDG‐PET/CT	—	Low‐grade sarcoma	+	POM	—	5 mm	+	24	—
18	Silva et al. [19]	2023	63	M	Maxilla	Buccal gingiva of molar region	100	Pedunculated	CBCT	—	N/A	—	POMF	—	None	—	12	—
19	Tatsis et al. [20]	2023	35	F	Mandible	Submucosa of the buccal mucosa in the molar region	30 × 10	Unilocular	CT	—	Lipoma or cyst	—	POM	+	Tumor capsule	—	N/A	N/A
20	Lillis et al. [21]	2023	66	F	Maxilla	Labial gingiva of anterior region	N/A	Pedunculated	N/A	N/A	Maxillary tumor	+	POMF	N/A	None	—	36	—
21	Nikunj et al. [22]	2024	16	M	Buccal region	From the lateral zygomatic region to the lateral orbital region	35 × 26	Unilocular	CT	—	Ameloblastoma	+	POM	+	Performed (details not available)	—	30	—
22	Tomar et al. [23]	2025	N/A	N/A	Maxilla	Labial gingiva of anterior region	20 × 10	Sessile	Panoramic radiograph	—	Traumatic fibroma	—	POM	—	None	N/A	36	—
23	Present case	2025	68	M	Maxilla	Palatal gingiva of molar region	8 × 4	Sessile	Panoramic radiograph and CT	—	Benign maxillary gingival tumor	—	POMF	+	5 mm	—	24	—


Abbreviations: CBCT, cone‐beam computed tomography; CT, computed tomography; FDG‐PET/CT, fluorodeoxyglucose–positron‐emission tomography–computed tomography; N/A, not applicable; POM, peripheral odontogenic myxoma; POMF, peripheral odontogenic myxofibroma; OPG, orthopantomography; OR, occlusal radiograph; PG, periapical radiograph; US, ultrasonography.

Intraosseous OM/OMF most frequently occurs in patients in their third to fourth decades of life, and pediatric cases are considered uncommon [24]. The mean age of patients with POM/POMF in the literature was 39 years (range, 8–68 years; median, 39.5), which is comparable with that of patients with intraosseous lesions. Although intraosseous OM/OMF is reported to occur more commonly in women and to show a predilection for the mandible over the maxilla (maxilla: mandible = 1 : 2), especially in the mandibular molar‐to‐ramus region [25], POM/POMF shows no obvious sex predilection and occurs with similar frequency in the maxilla and mandible. From a practical standpoint, this distribution means that clinicians should consider POM/POMF in both arches when evaluating persistent gingival masses, particularly along the marginal gingiva in tooth‐bearing areas. Rare cases have also been reported outside the gingiva, such as in the infratemporal fossa and cheek regions.

POMF is an odontogenic mesenchymal tumor, and its presumed origin is odontogenic ectomesenchyme derived from the developing dental papilla, dental follicle, or periodontal ligament [2, 26, 27]. The frequent occurrence of these tumors in tooth‐bearing areas of the jaws and the presence of inactive islands or strands of odontogenic epithelium in some cases support this odontogenic origin [2, 3, 26]. Tumor cells are stellate to spindle‐shaped and are thought to represent fibroblasts or myofibroblasts. These primitive mesenchymal cells have been proposed to produce excessive amounts of acidic mucopolysaccharides with a relatively reduced capacity to generate mature collagen, thereby creating a fibromyxoid pattern in which myxoid and fibrous stroma coexist [2, 28]. This characteristic composition explains the soft, gelatinous appearance described for many intraosseous lesions and also accounts for the hypocellular, myxoid stroma observed in peripheral lesions.

Clinically, POMF must be differentiated from other gingival masses, such as peripheral giant cell granuloma, pyogenic granuloma, peripheral ossifying fibroma, odontogenic fibroma, and epulis [11, 21]. Because these reactive or inflammatory lesions are far more common in daily practice, clinicians may reasonably prioritize them at first presentation; however, persistence, progressive enlargement, or atypical consistency should prompt reconsideration and tissue diagnosis to exclude rare entities such as POM/POMF. In practice, the differential diagnosis also includes minor salivary gland lesions and other benign soft‐tissue tumors depending on the site and surface changes. However, no imaging modality is specifically useful for diagnosing POM/POMF, and histopathological examination is therefore crucial. In OMF, stellate to spindle‐shaped cells are sparsely distributed within an abundant myxoid stroma rich in acidic mucopolysaccharides, with varying amounts of intermingled collagen fibers; cytologic atypia and mitotic figures are rare [2, 28, 29]. Special stains typically show positivity for Alcian blue and negativity for periodic acid–Schiff (PAS), and reticulin staining often reveals a fine network of fibers [28, 29]. Histologically, OMF must be differentiated from other myxoid lesions including soft‐tissue myxoma, nerve sheath myxoma, myxoid neurofibroma, and oral focal mucinosis [3, 28]. In the present case, the presence of an Alcian blue–positive myxoid stroma with scattered odontogenic epithelial nests supported the diagnosis of POMF and helped exclude nonodontogenic myxoid entities.

From a diagnostic‐workup standpoint, cross‐sectional imaging is primarily useful to assess whether the lesion is truly extraosseous and to evaluate potential cortical contact or superficial erosion. When CT/CBCT demonstrates no bone involvement, the lesion is more likely to be peripheral, and a jaw‐preserving procedure can be planned. Conversely, radiographic aggressiveness or ulceration/rapid growth should raise concern for alternative diagnoses and favor incisional biopsy before definitive surgery. In addition, exfoliative cytology can be used as an adjunct to evaluate the epithelial surface and may help exclude epithelial malignancy, but it does not provide a definitive diagnosis for mesenchymal odontogenic tumors; therefore, tissue diagnosis remains indispensable.

From a clinical perspective, a key pitfall is that POM/POMF lesions often lack lesion‐specific radiographic changes and may be indistinguishable from common reactive gingival lesions in tooth‐bearing areas. In our case, the lesion was initially diagnosed as chronic periodontitis at a local clinic, and panoramic radiography at presentation demonstrated only mild generalized horizontal bone loss without lesion‐specific changes. This pitfall is amplified in tooth‐bearing areas because baseline periodontal bone loss can mask the absence of lesion‐specific radiographic changes, encouraging clinicians to interpret the swelling within a periodontal framework. In our case, the combination of (i) a localized marginal gingival mass adjacent to a tooth, (ii) persistence over 6 months, (iii) elastic–firm consistency with partial erythema, and (iv) absence of bone involvement on CT supported an extraosseous process and prompted excisional biopsy for definitive diagnosis. Therefore, a persistent, localized gingival swelling in a tooth‐bearing area—particularly when it persists despite observation or periodontal management—should prompt cross‐sectional imaging to assess bone involvement and timely biopsy or excisional biopsy for histopathological confirmation. Excisional biopsy can be reasonable for small, superficial lesions with low suspicion of malignancy, whereas incisional biopsy is preferable for lesions that are large, ulcerated, rapidly growing, or radiographically aggressive. This practical framework is aimed at preventing delayed recognition and incomplete initial excision, which may contribute to recurrence.

Immunohistochemically, tumor cells are reported to be positive for vimentin and negative for S100, CD34, and SMA, findings that are useful for excluding nerve sheath, vascular, and myogenic myxoid lesions [27, 30–32]. Odontogenic epithelial islands are positive for cytokeratins, including CK19, thus providing supportive evidence of an odontogenic origin [27, 31]. Although these immunoprofiles are not pathognomonic, they are useful as a panel to support the diagnosis in conjunction with morphology and special stains and to exclude major myxoid mimics. Small nests or strands of odontogenic epithelium may be scattered within the tumor; however, their detection rate is generally low, and they are not considered essential for histopathological diagnosis [33]. In the present review, odontogenic epithelium was identified in 9 of 22 previously reported POM/POMF cases (40.9%). When odontogenic epithelium is demonstrable, the odontogenic origin becomes unequivocal and can be particularly helpful when the lesion is small or superficially located. In our case, the demonstration of odontogenic epithelium together with Alcian blue–positive myxoid stroma contributed to the final diagnosis.

Treatment options for intraosseous OM/OMF depend on tumor size and include jaw‐preserving procedures (simple tumor excision or excision with peripheral ostectomy) and radical surgery with a 10–15 mm safety margin. The recurrence rate of intraosseous OM/OMF has been reported to be 25%–43%, with most recurrences occurring within 2 years after surgery [34]. In contrast, no consensus has been reached regarding the optimal treatment for POM/POMF, and jaw‐preserving excision has generally been preferred in previously reported cases. The reported recurrence rate for POM is 3%–8% [13]. Among the cases treated in oral and maxillofacial surgery units, no recurrences were observed during follow‐up periods of 6–108 months (mean, 32.5). However, Cases 5, 9, and 16 in our review were initially excised at local dental clinics under a clinical diagnosis of fibroma or epulis and subsequently recurred, suggesting that incomplete excision contributed to recurrence. These observations support securing an adequate soft‐tissue margin (potentially including the periosteum) at the initial procedure. Selective bone removal, such as limited peripheral ostectomy, may be considered when there is suspected cortical contact, when margin control is needed, or when superficial cortical involvement cannot be excluded based on intraoperative findings. In our patient, complete excision with a 5‐mm clinical margin including the periosteum and selective limited peripheral ostectomy resulted in uneventful healing and no recurrence at 2 years, consistent with the jaw‐preserving strategies reported for most POM/POMF cases in Table 2. Given that recurrences of intraosseous lesions tend to occur within 2 years [34], careful follow‐up is also warranted for peripheral lesions, particularly in cases initially treated under an incorrect diagnosis.

In the present case, no recurrence was observed during a 2‐year postoperative follow‐up period. Overall, this report is intended primarily as an educational case report highlighting a specific diagnostic pitfall—misattribution of a persistent localized gingival mass to periodontal disease in the setting of nonspecific radiographic findings—and providing practical diagnostic and surgical considerations for persistent gingival masses in tooth‐bearing areas rather than as evidence of a fundamentally new disease entity or treatment concept.

## Author Contributions

Masanori Masui: conceptualization, investigation, data curation, writing—original draft, and visualization. Hirokazu Yutori: writing—review and editing. Ai Fujimura: investigation, resources, and writing—review and editing. Soichiro Ibaragi: supervision and writing—review and editing.

## Funding

No funding was received for this manuscript.

## Disclosure

The paper was presented in “The 70th General Meeting and Scientific Congress of the Japanese Society of Oral and Maxillofacial Surgeons.”

## Ethics Statement

This case report was determined to be exempt from institutional ethics committee review according to our institutional policy because it describes a single patient and contains no identifiable personal information. The report was prepared in accordance with the principles of the Declaration of Helsinki.

## Consent

Written informed consent was obtained from the patient for publication of this case report and the accompanying images.

## Conflicts of Interest

The authors declare no conflicts of interest.

## Supporting information


**Supporting Information** Additional supporting information can be found online in the Supporting Information section. File S1: CARE checklist for this case report.

## Data Availability

All data related to the present case are included in this published article.
