# Supplementary material for: A Case of Peripheral Odontogenic Myxofibroma Arising in the Palatal Gingiva of the Maxillary Second Premolar Region: A Case Report
Source: Case Rep Dent. 2026 Jun 4;2026:7333467. doi: 10.1155/crid/7333467 (PMC13238269; doi:10.1155/crid/7333467)
Supplement: Supplementary file 1 — Supporting Information Additional supporting information can be found online in the Supporting Information section. File S1: CARE checklist for this case report. [file CRID-2026-7333467-s001.zip › 7333467.f1/4_CARE checklist.pdf]

CARE case report guidelines

CARE Checklist of information to include when writing a case report

(cc) BY-NC-ND

Topic	Item	Checklist item description	Reported on Line
Title	1	The diagnosis or intervention of primary focus followed by the words "case report"	p.1, lines 1-2
Key Words	2	2 to 5 key words that identify diagnoses or interventions in this case report, including "case report"	p.2, lines 25-26
Abstract (no references)	3a	Introduction: What is unique about this case and what does it add to the scientific literature?	p.2, lines 2-6
	3b	Main symptoms and/or important clinical findings	p.2, lines 2-11
	3c	The main diagnoses, therapeutic interventions, and outcomes	p.2, lines 11-20
	3d	Conclusion—What is the main "take-away" lesson(s) from this case?	p.2, lines 20-24
Introduction	4	One or two paragraphs summarizing why this case is unique (**may include references**)	p.3, lines 1-10
Patient Information	5a	De-identified patient specific information.	p.3, lines 12
	5b	Primary concerns and symptoms of the patient.	p.3, lines 12-13, 16-22
	5c	Medical, family, and psycho-social history including relevant genetic information	p.3, lines 13-15
	5d	Relevant past interventions with outcomes	p.3, lines 16-19
Clinical Findings	6	Describe significant physical examination (PE) and important clinical findings.	p.3, lines 20-22
Timeline	7	Historical and current information from this episode of care organized as a timeline	p.4, lines 16–17, Table 1
Diagnostic Assessment	8a	Diagnostic testing (such as PE, laboratory testing, imaging, surveys).	p.3, lines 23–26, 27-29,p.4, lines 10-17
	8b	Diagnostic challenges (such as access to testing, financial, or cultural)	N/A
	8c	Diagnosis (including other diagnoses considered)	p.3, lines 25–26, p.5, lines 20–22
	8d	Prognosis (such as staging in oncology) where applicable	N/A
Therapeutic Intervention	9a	Types of therapeutic intervention (such as pharmacologic, surgical, preventive, self-care)	p.4, lines 4–7
	9b	Administration of therapeutic intervention (such as dosage, strength, duration)	p.4, lines 4–7
	9c	Changes in therapeutic intervention (with rationale)	N/A
Follow-up and Outcomes	10a	Clinician and patient-assessed outcomes (if available)	p.4, lines 7–9
	10b	Important follow-up diagnostic and other test results	p.4, lines 8–9
	10c	Intervention adherence and tolerability (How was this assessed?)	N/A
	10d	Adverse and unanticipated events	p.4, line 7
Discussion	11a	A scientific discussion of the strengths AND limitations associated with this case report	p.4, line 18 ～ p.6, line 25
	11b	Discussion of the relevant medical literature **with references**.	p.4, line 18 ～ p.6, line 23
	11c	The scientific rationale for any conclusions (including assessment of possible causes)	p.6, lines 23–30
	11d	The primary "take-away" lessons of this case report (without references) in a one paragraph conclusion	p.6, lines 24–25
Patient Perspective	12	The patient should share their perspective in one to two paragraphs on the treatment(s) they received	Not reported.
Informed Consent	13	Did the patient give informed consent? Please provide if requested	Yes ☑ No ☐
