# Supplementary material for: A Case of Peripheral Odontogenic Myxofibroma Arising in the Palatal Gingiva of the Maxillary Second Premolar Region: A Case Report
Source: Case Rep Dent. 2026 Jun 4;2026:7333467. doi: 10.1155/crid/7333467 (PMC13238269; doi:10.1155/crid/7333467)
Supplement: Supplementary file 1 — Supporting Information Additional supporting information can be found online in the Supporting Information section. File S1: CARE checklist for this case report. [file CRID-2026-7333467-s001.zip › 7333467.f1/Certificate_of_editing-GVPMJ_1_xyeasv9plk.pdf]

editage

Editing Certificate

Issued On March 17, 2026

This document certifies that the manuscript listed below has been partially edited (excluded section/s: Title) to ensure language and grammar accuracy and is error free in these aspects. The edit was performed by professional editors at Editage, a brand of Cactus Communications. The author's core research ideas were not altered in any way during the editing process. The quality of the edit has been guaranteed, with the assumption that our suggested changes have been accepted and the text has not been further altered without the knowledge of our editors.

Manuscript Title

A Case of Peripheral Odontogenic Myxofibroma Arising in the Palatal Gingiva of the Maxillary Second Premolar Region: A Case Report

Authors

Masanori Masui

GVPMJ_1

Job Code

Prabh Grewal, Senior Vice President, Editage

QUALITY GUARANTEE • QUALITY GUARANTEE • QUALITY GUARANTEE •
THE editage PROMISE

VERIFY AT
https://editage.jp/ecverify

VERIFICATION CODE
EC-260317-TQFLN7
